# Solid-State Conversion of Magnesium Waste to Advanced Hydrogen-Storage Nanopowder Particles

**DOI:** 10.3390/nano10061037

**Published:** 2020-05-28

**Authors:** Mohamed Sherif El-Eskandarany, Naser Ali, Sultan Majed Al-Salem

**Affiliations:** 1 Energy and Building Research Center, Kuwait Institute for Scientific Research, Safat 13109, Kuwait; nmali@kisr.edu.kw; 2 Environment & Life Sciences Research Centre, Kuwait Institute for Scientific Research, Safat 13109, Kuwait; ssalem@kisr.edu.kw

**Keywords:** solid waste, magnesium, milling, metal waste, magnesium hydride

## Abstract

Recycling of metallic solid-waste (SW) components has recently become one of the most attractive topics for scientific research and applications on a global scale. A considerable number of applications are proposed for utilizing metallic SW products in different applications. Utilization of SW magnesium (Mg) metal for tailoring high-hydrogen storage capacity nanoparticles has never been reported as yet. The present study demonstrates the ability to produce pure Mg ingots through a melting and casting approach from Mg-machining chips. The ingots were used as a feedstock material to produce high-quality Mg-ribbons, using a melting/casting and spinning approaches. The ribbons were then subjected to severe plastic deformation through the cold rolling technique. The as-cold roll Mg strips were then snipped into small shots before charging them into reactive ball milling. The milling process was undertaken under high-pressure of pure hydrogen gas (H_2_), where titanium balls were used as milling media. The final product obtained after 100 h of milling showcased excellent nanocrystalline structure and revealed high hydro/dehydrogenation kinetics at moderate temperature (275 °C). The present study shows that primer cold rolling of Mg-strips before reactive ball milling is a necessary step to prepare ultrafine magnesium hydride (MgH_2_) nanopowders with advanced absorption/desorption kinetics behavior. These ultrafine powders with their nanocrystalline structure are believed to play an important role in effective gas diffusion process. Moreover, the fine titanium particles came from the ball-powder-ball collisions and introduced to the Mg matrix have not only acted as micro-scaled milling media, but they played a vital catalyzation role for the process.

## 1. Introduction

### 1.1. Background

#### 1.1.1. Solid-Waste Management

Historically, waste is stigmatized as an undesired material, which must be disposed of after its primary use. It was believed that solid waste (SW) management is a difficult and expensive task that requires prolonged time-consuming procedures. In the present day, significant strides are achieved in dealing with SW components of various types to overcome concerns with their recycling and recovery procedures [1,2]. SW of metallic origin and alloys is a prime example of it originating from scrap automobiles and the industrial sector [3,4,5,6]. Global interest in recycling ferrous and nonferrous metal alloys including magnesium (Mg) metal, has received great attention as of late. This is due to their favorable engineering properties [7,8,9]. Its lightness, excellent flexibility and abundance as a natural resource, make Mg a particularly desired material, especially when the overall strength of a material is desired [10]. 

#### 1.1.2. Nano-Mg as a Solid-State Hydrogen Storage Materials

In addition to the useful applications of Mg in a wide range of industrial sectors (e.g., automotive, aerospace, medical, and electronics applications) it has been considered as a promising candidate material for solid-state hydrogen storage applications [11,12,13,14]. Metallic Mg possesses a unique combination of high gravimetric (7.60 wt%) and volumetric (110 g/L) hydrogen storage capacities, as well as a good cyclability [15]. It should be noted that tetragonal magnesium hydride (MgH_2_) in *β*-phase is a very stable compound in a thermodynamic sense, requiring elevated temperatures to decompose (>350 °C) [14]. It also exhibits a high value of apparent activation energy (E_a_ > 130 kJ/mol) [16]. Below this temperature, MgH_2_ shows very slow hydrogenation/dehydrogenation kinetics [17]. 

For MgH_2_ to be employed in fuel cell applications, it must undergo severe treatment processes dedicated to accelerating its inherently poor kinetic behavior, in order to reduce its activation energy of decomposition [18,19,20]. Within the last three decades, different options were proposed to improve the hydrogen storage behavior of Mg/MgH_2_. These options include severe plastic deformation (SPD), high-energy ball milling (HEBM), cold-rolling (CR), equal channel angular pressing (ECAP), and high-pressure torsion (HPT) [21,22,23]. 

Doping Mg/MgH_2_ powders with catalytic agents is a different approach used to increase the hydrogenation/dehydrogenation kinetic of MgH_2_. Since the 1990s, a wide range of pure transition metals (TM) have been used at different concentrations to improve the behavior of Mg/MgH_2_ powders. Due to their superior capability for hydrogen splitting (dissociation) and re-combination, many of these metals exerted remarkable beneficial effects in modifying hydrogen storage properties of Mg/MgH_2_. Elemental nickel (Ni), titanium (Ti), vanadium (V), and manganese (Mn) metals [24,25], as well as their alloys [26,27,28,29,30] were successfully employed to improve the hydrogen storage behavior of MgH_2_ powders. In addition to the metals and their alloys, compounds and metastable catalytic agents, metal-oxides (e.g., Nb_2_O_5_ [31,32], Cr_2_O_3_ [33], and TiO_2_ [34], -carbides (e.g., SiC [35], and TiC [36]), -hydrides (e.g., TiH_2_ [37], LaH_3_ [38], and NbH [38]), and carbon-based nanomaterials such as carbon nanotubes [39], and graphene nanofibers [40]. 

### 1.2. Aim of the Present Study

The present study focuses in part on the possibility of utilizing SW-Mg machining chips with the aim of fabricating high-quality Mg nanopowder particles, using a multistage process of melting/casting, melt spinning (MS) and CR. The effect of the plastic deformation generated by CR on the grain size of Mg-ribbons was studied as a function of the number of passes, in the range between 0 to 150 passes. The as-refined pure Mg-metal was used as feedstock materials for synthesizing nanocrystalline MgH_2_ powders through a reactive ball milling (RBM) approach. Moreover, this study has investigated the effect of using pure Ti-ball milling media on the hydrogenation/dehydrogenation kinetics of MgH_2_ upon milling for different RBM times. To the best of our knowledge, there exist various studies that show different methods of recycling Mg [7,8,9], but no studies report as of yet the possibility of preparing Mg/MgH_2_ nanopowder particles from an SW feedstock. 

## 2. Materials and Methods 

### 2.1. The Feedstock Materials

**Figure 1:**
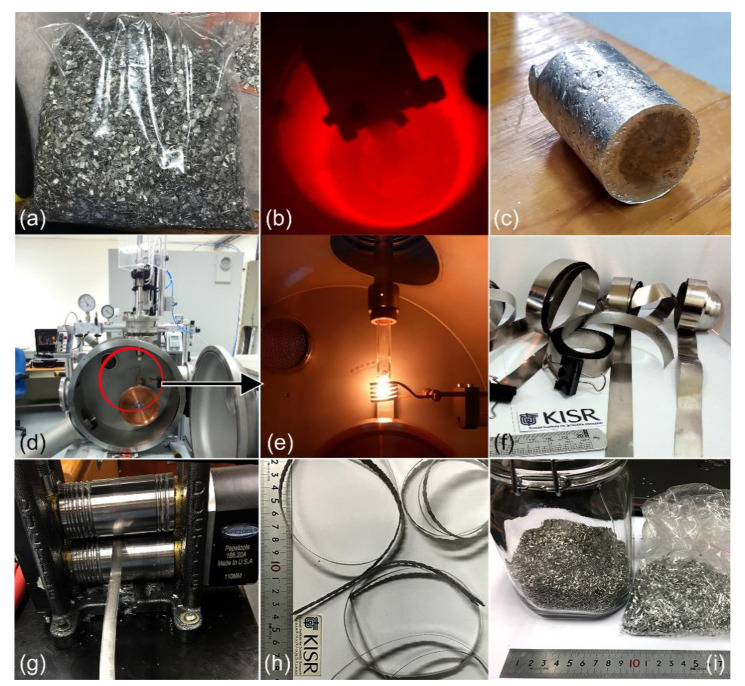
Conversion of solid-waste (SW) Mg machining chips into microscaled Mg-shots. (**a**) as-received SW-Mg chips, (**b**) melting and casting processes, (**c**) as-casted pure (99.95 wt%) Mg-ingot, (**d**), (**e**) melt spinning (MS) process, (**f**) as-MS Mg-ribbons, (**g**) cold rolling (CR) process, (**h**) as-CR Mg-strips, and (**i**) as-snipped Mg-shots.

For the purpose of the present study, a batch of 10 kg of pure (99.5 wt%) Mg chips (25–35 mm in length and ~20 mm in thickness) were acquired from Shanghai Xinglu Chemical Technology Co., Ltd., Shanghai, China, and used as feedstock materials (Figure 1a). The received chips were firstly sonicated in a cold-acetone bath for 10 min to ensure removing all machining oil coolants from their surfaces. The chips were then rinsed with pure ethanol before drying in an oven at 180 °C for a continuous period of 18 h. This primer-treating step was necessary to remove all of the undesired hydrocarbon contaminants from the chip’s surfaces. A 300 g amount of oil-free chips was then placed in a graphite crucible and placed in a conventional melting/casting machine (Figure 1b). 

The induction melting section was firstly evacuated to the level of 10^−5^ bar before being filled with pure argon (Ar, 99.99 wt%) gas. Then, the melting process was performed at 750 °C for three continuous cycles. During the melting process, 100 mL of Ar gas was introduced frequently, in order to ensure the purification of the molten Mg. Towards the end of the melting procedure, the bottom part of the graphite crucible was opened, where the molten metal was sunk into a cylindrical graphite crucible at a temperature of almost 400 °C. The system was kept for 9 h before opening it to remove the Mg ingot from the crucible (Figure 1c). The elemental analysis of the cast-Mg indicated that the material was ultra-pure (99.88 wt%) with less than 0.11 wt% oxygen and 0.01 wt% carbon, as characterized by inert gas fusion, and thermal conductivity detection test techniques, respectively. 

About 5 g of small pieces (~30–40 mm^3^) of the as-cast Mg ingot were inserted in a quartz tube and fixed in PA 500 melt spinner (MS) machine, provided by Edmund Bühler, Germany (Figure 1d). A single Cu-wheel drum, rotated at 5000 rpm was used to provide a rapid quenching of the melt. The MS process was first evacuated to the level of 10^−6^ before introducing pure helium (99.99 wt%) gas to the quartz-crucible and MS-chamber. Then, the crucible was pressurized with 3 bar of He gas, and the chamber was maintained under 0.8 bar of He. The induction melting was taken place at 750 °C (Figure 1e). The molten Mg-fluid was forced to travel into the hole in the bottom of the quartz-crucible upon pressurizing the crucible with an extra 2 bar of He gas. The Mg-droplets passed from the hole were rapidly quenched with a very high cooling rate generated by the rotated Cu-drum. The end product of MS-Mg was in the shape of ribbons of ~0.5 mm in thickness, and ~300 cm in length, as shown in Figure 1f. 

The as-MS ribbons were cut into smaller strips of almost 20 cm in length and then subjected to CR for 150 passes, using a conventional two-tool steel drum cold roller (Figure 1g). The strips were warm pressed at 150 °C for 5 min after every 10 passes using a two -plate warm press to avoid brittleness in the Mg strips that occurred during the CR process. The as-CR Mg strips were elongated by approximately 112% after the completion of 150 passes (Figure 1h). The average thickness of the as-CR Mg strips was reached to 108 μm after CR. The CR Mg-strips were cut into short ribbons of approximately 4 to 10 mm in length, 0.5 cm in thickness. These snipped strips were simply cleaned with acetone and ethanol, and then dried in an oven at 150 °C overnight. The as-snipped Mg shots were sealed under He gas atmosphere and kept in an argon glove box (Figure 1i).

### 2.2. Preparation of MgH_2_ Nanopowder Particles

**Figure 2:**
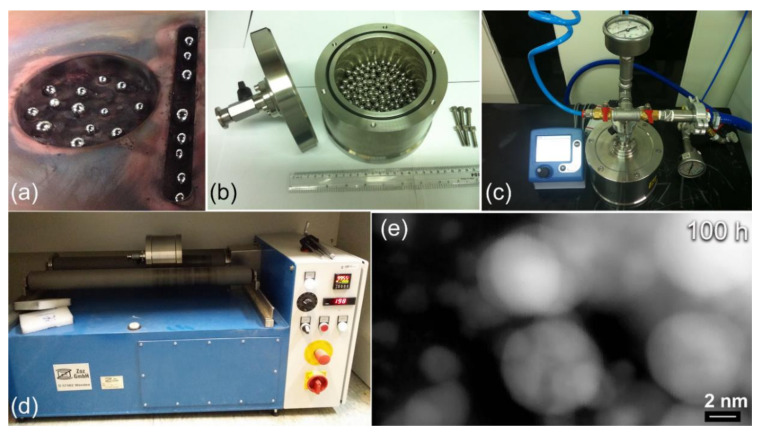
Reactive ball milling (RBM) process, employed for preparing nanocrystalline MgH_2_ powder particles. (**a**) Preparing of Ti balls milling media with arc melting process, (**b**) tool-steel milling vial, charged with Mg-shots, (**c**) the milling vial was pressurized with 15 bar of hydrogen gas, and (**d**) the RBM process was carried out at room temperature, using a roller mill. The scanning transmission electron (STEM) image of CR MgH_2_-ribbons obtained after 100 h of RBM is displayed in (**e**).

Figure 2 summarizes the experimental procedure performed for preparing nanodimensional MgH_2_ particles, using the RBM technique. We firstly prepared a large number of pure Ti-balls using an arc melter (Edmund Bühler, Germany). In this process, sponge Ti was melted under pure He (99.99 wt%) gas atmosphere in a Cu-hearth. The Ti pieces were then remelted again in the arc melter to produce nearly spherical balls with diameters of 11 mm to 13 mm, as shown in Figure 2a. 

An amount of 50 g of Mg shots and a number of 250 Ti-balls were charged into a cylindrical vial made of tool-steel and sealed together in a glove box (UNILAB Pro Glove Box Workstation, mBRAUN, Germany) under pure (99.99 wt%) He gas atmosphere, as shown in Figure 2b. The ball-to-powder weight ratio was 36:1. The sealed vial was then evacuated to the level of 10^−3^ bar or better before pressurized with 15 bar of pure (99.999 wt%) H_2_ gas, as displayed in Figure 2c. 

The vial was mounted on the roller of a tumbling mill, rotated at 300 rpm (Figure 2d). The RBM process was conducted at room temperature, using a milling speed of 250 rpm. To understand the mechanism of mechanically-induced gas-solid reaction taking place during the RBM process, the tumbling mill was interrupted after selected milling time (3, 6, 12.5, 18, 25, 37.5, 50, 100, and 150 h), where the milled powders were completely discharged from the vial in the He-glove box. The RBM process was resumed with fresh batches of CR Mg-strips, using the same milling parameters. The scanning transmission electron (STEM) image of the powders for the end-product sample (100 h of RBM) is displayed in Figure 2e. The powders after this final milling stage composited of nanoscaled spherical particles, with particle size laid in the range from ~2 nm to 6 nm in diameter, as shown in Figure 2e.

### 2.3. Sample Characterizations

#### 2.3.1. Crystal Structure

The crystal structures of all samples were investigated by X-ray diffraction (XRD) with CuKα radiation, using 9 kW Intelligent X-ray diffraction system, provided by SmartLab (Rigaku, Japan). The local structure of the synthesized materials was studied by 200 kV-field emission high-resolution transmission electron microscopy/scanning transmission electron microscopy (HRTEM/STEM) supplied by JEOL-2100F (Tokyo, Japan), and equipped with energy-dispersive X-ray spectroscopy (EDS) supplied by Oxford Instruments, (Abingdon, UK). A Cryo Ion Slicer Machine (IB-09060CIS) supplied by JEOL-2100F, (Tokyo, Japan) was used to prepared TEM samples of as-CR Mg strips.

#### 2.3.2. Morphology and Elemental Analysis

The morphological characteristics of the milled and consolidated samples were investigated by means of field-emission scanning electron microscope (FE-SEM), using 15 kV- JSM-7800F, JEOL, (Tokyo, Japan). The local elemental analysis was investigated by EDS (Oxford Instruments, Abingdon, UK) system interfaced with the FE-SEM.

#### 2.3.3. Thermal Stability

Differential scanning calorimeter (DSC), provided by Setaram Instrumentation (Caluire, France)—was employed to investigate the decomposition temperature of MgH_2_ powder samples that were obtained after different RBM time.

#### 2.3.4. Hydrogenation/Dehydrogenation Kinetics Behavior

The absorption/desorption kinetics of MgH_2_ powders obtained after different RBM times were investigated at 275°C by means of Sievert’s method, using PCT-Pro2000 (Setaram Instrumentation, Caluire, France). The applied hydrogenation and dehydrogenation pressures were 10 bar and 400 mbar, respectively.

## 3. Results and Discussion

### 3.1. Morphology and Crystal Structure 

#### 3.1.1. CR Mg-Ribbons

**Figure 3:**
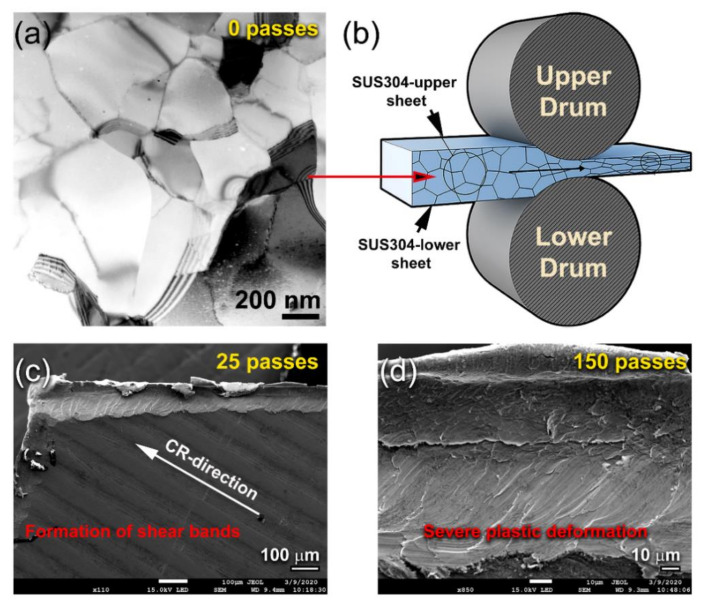
(**a**) Low magnification transmission electron microscopy (TEM)-BFI of as-MS Mg-ribbon, and (**b**) schematic illustrates the CR process of Mg-chips. The low magnification field-emission scanning electron microscope (FE-SEM) micrographs of Mg-ribbons after CR for 25 and 150 passes are displayed in (**c**) and (**d**), respectively.

**Figure 4:**
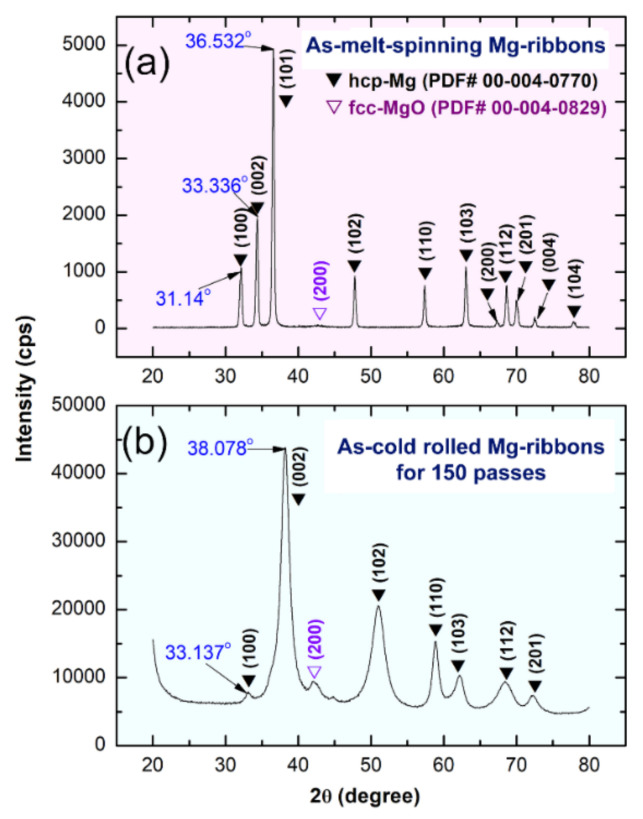
X-ray diffraction (XRD) patterns of Mg-ribbons after CR for (**a**) 0 passes, and (**b**) 150 passes.

TEM image in a low-magnification bright field (BF) mode for the planner view of a polished sample of as-MS Mg ribbon is displayed in Figure 3a. The sample composited of a mixture of large- and medium-sized grains, with an average grain size of ~480 nm (Figure 3a). Initially, Mg-ribbons revealed twin free-grain boundaries with no indication latter of lattice imperfections (Figure 3a). The XRD pattern of the as-MS Mg-ribbons is displayed in Figure 4a. The sample exhibited sharp Bragg peaks corresponding to hcp-Mg (PDF 00-004-0770) without evidence of any Bragg-peaks’ mismatches (Figure 4a). This could be attributed to the absence of lattice imperfections in the MS-Mg ribbons. 

**Figure 5:**
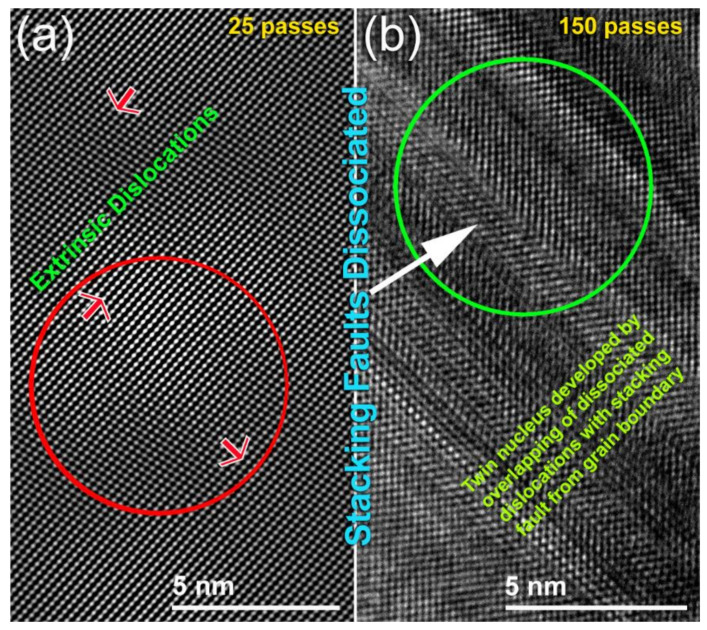
Atomic resolution FE-TEM images of Mg-ribbons after CR for (**a**) 25 passes, and (**b**) 150 passes.

When the ribbons were cold-rolled for 25 passes, they revealed obvious micro-intimated bands that have developed as a result of the shear stresses applied to them during the CR process (Figure 3c). This was confirmed by the BF-TEM image of a cross-section view of the as-CR Mg ribbons obtained after 25 passes that revealed lattice imperfections beyond the atomic level, as indexed by the extrinsic dislocations presented in Figure 5a. Severe plastic deformation was realized upon increasing the CR passing time to 150. This is implied by the formation of ultrafine bent shear bands with less than 5 μm in width, as displayed in Figure 3d. In contrast to the initially studied MS-Mg ribbons, two major hcp-Mg Bragg peaks, namely, (100) and (002), were shifted to the high-angle side, where the major crystallographic plane (101) had completely disappeared in the sample obtained after 150-passes of CR (Figure 4b). This implies a severe lattice imperfection conducted during the CR process [41] and the development of a very high level of the plane (002) of fiber texture [42]. The significant broadening that observed in the Bragg peaks for the sample obtained after 150 passes of CR (Figure 4b) was attributed to the internal strain related to the existence of a high dislocation density and grain refinement. 

After this final stage of CR (150 passes), the sample possessed twin nuclei that overlapped with dissociated dislocation and stacking faults from the grain boundary, as as seen in Figure 5b. This could be explained by the deformation that developed in hcp-metals which are typically accommodated by twinning and basal slip deformation mechanisms [43]. Moreover, the CR ribbons exhibited different types of twins, such as V-shaped, and deformation twins, as illustrated in Figure 5b. Accordingly, this twinning that developed during the CR process is believed to be responsible for the formation of different amounts of (002) texture, as suggested by Jorge et al. [42].

#### 3.1.2. Reactive Ball Milling of Mg Powders 

**Figure 6:**
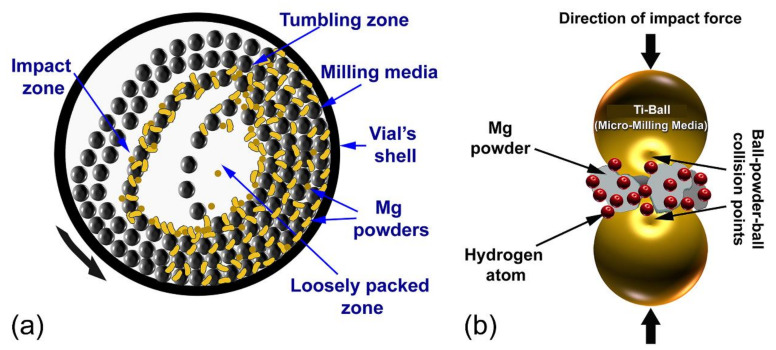
Schematic presents the (**a**) movement of the balls and materials (Mg shots and powders) inside a sealed cylindrical vial, which is pressurized with 15 bar of H_2_ gas, and (**b**) ball-powder-ball collision during RBM process, using a tumbler ball mill.

**Figure 7:**
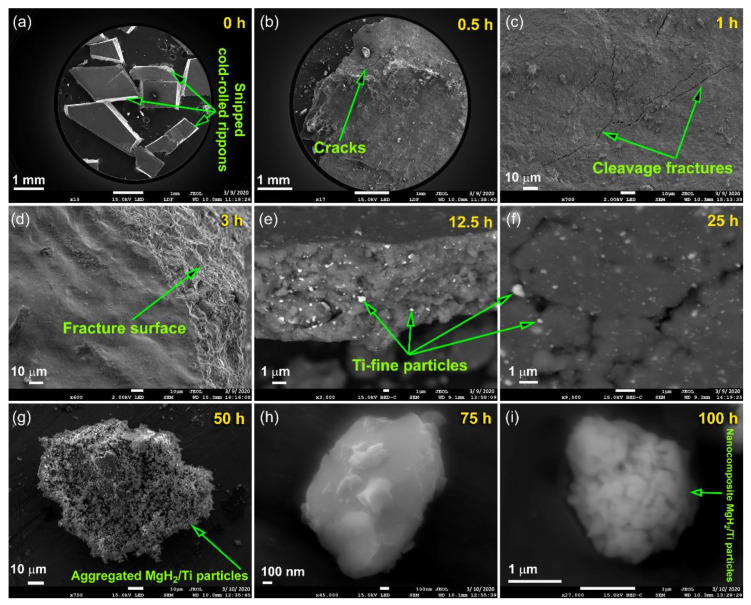
FE-SEM images of Mg-strips obtained after CR for 150 passes and then RBM for (**a**) 0, (**b**) 0.5, (**c**) 1, (**d**) 3, (**e**) 12.5, (**f**) 25, (**g**) 50, (**h**) 75, and (**i**) 100 h of RBM time.

**Figure 8:**
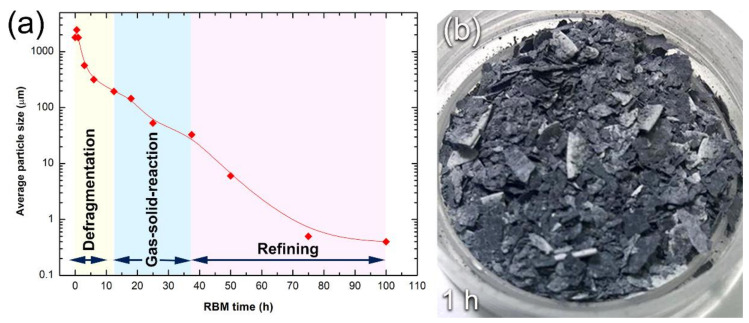
(**a**) Particle size distribution of as-CR Mg strips during the different stages of milling named; defragmentation; gas-solid reaction; and refining stages, and (**b**) an optical image of the sample obtained after 0.5 h RBM.

Figure 6a depicts a schematic illustration of the tumbler ball mill, used in this work to perform a solid-state hydrogenation reaction between Mg and hydrogen gas. In the reactive ball milling (RBM) process, the starting CR Mg-shots, which are composed of large pieces (500 μm to 2000 μm) (Figure 7a) are in contact with the abrasive forces generated by the Ti balls—milling media (Figure 6a,b). After 30 min of the RBM process, the Mg metal-pieces tended to agglomerate into larger pieces, have almost 3500 μm in size (Figure 7b or Figure 8a). The agglomeration, which was happened during the first stage of RBM was attributed to the successive cold-welding of the ductile Mg-metal [44]. This resulted from the impact forces generated upon balls-particles-ball collision, as illustrated in Figure 6b. 

The useful kinetic energy in tumbling mill applied to the Mg-shots can be summarized by the following means (1) collision between the balls and the particle; (2) pressure loading of particles pinned between milling media or between the milling media and the liner; (3) impact of the falling milling media; (4) shear and abrasion caused by dragging of particles between moving milling media; and (5) shock-wave transmitted through crop load by falling milling media [45]. 

In general, the RBM process for the formation of MgH_2_ powder particles can be classified into three consequent stages; (1) defragmentation; (2) gas-solid reaction; and (3) refining stages, as presented in Figure 8a. After 1 h of RBM time, the impact led to deform the ductile Mg-particles to yield aggregates with plate-like morphology, as presented in Figure 7c. However, the sample obtained after this stage of milling revealed wide size distribution, extended from a few mm to several mm in size, as shown in Figure 8b. Application of the impact forces to the particles led to increasing their hardness due to work hardening. Accordingly, the Mg-particles became brittle, as indicated by the cracks and cleavage fractures indexed in Figure 7c. Further RBM time (3 to 12.5 h) led to severe fragmentation of Mg-particles, where the MgO-crust coated the particles were gradually removed with increasing the RBM time, as presented in Figure 7d,e. This milling step led to creating fresh-Mg surfaces ready to react with hydrogen milling atmosphere [46]. We should emphasize that the fine Ti-particles indexed in Figure 7e resulted from the erosion that took place between Ti balls (milling media) during the milling process. Such fine micro-milling media are believed to play a vital role in reducing the Mg particle size and to enhance the hydrogenation reaction during the RBM process [47]. 

**Figure 9:**
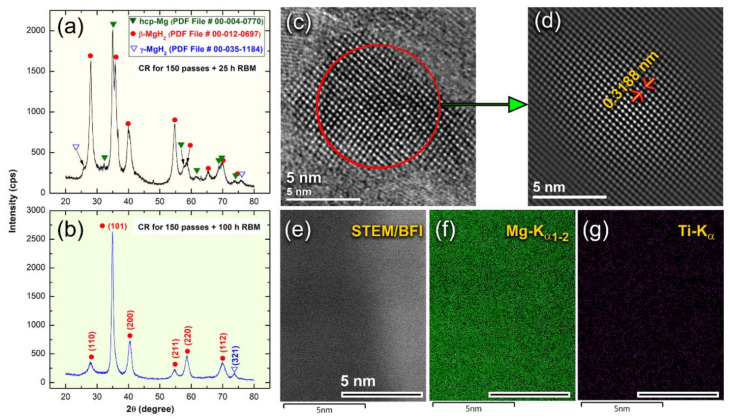
XRD patterns of Mg-ribbons obtained after CR for 150 passes then RBM for (**a**) 25, and (**b**) 100 h of RBM time. The high-resolution transmission electron microscopy/scanning transmission electron microscopy (HRTEM) image of the powder particles obtained after 100 h of RBM is displayed in (**c**), where a filtered atomic-resolution TEM image taken from the indexed zone shown in (**c**) is presented in (**d**). Elsewhere, the STEM/BFI image of the powder obtained after 100 of RBM is displayed in (**e**) together with the related X-ray energy-dispersive X-ray spectroscopy (EDS) elemental analysis of (**f**) Mg and (**g**) Ti.

Further RBM time (25 h) led to performing significant disintegration of the brittle Mg-powders (Figure 7f) to obtain finer particles (Figure 7f) with sizes ranging from 5 to 65 μm in diameter, as presented in Figure 8a. At the second stage of milling (gas-solid reaction stage), the XRD patterns of the sample obtained after 25 h of RBM revealed a new set of Bragg-peaks (Figure 9a) that could not be identified for the sample obtained after 150 passes of CR (Figure 4b). The analysis of these new diffraction lines indicated that the sample obtained after 25 h of RBM time composited of β-MgH_2_, γ-MgH_2_, as well unreacted hcp-Mg metal, and phases, as displayed in Figure 9a. 

After 50 h of continuous RBM, microscaled MgH_2_ powder particles (~8 μm) were obtained, as shown in Figure 7g or Figure 8a. These formed fine particles tended to be agglomerated due to van der Waals forces to form larger aggregates with apparent sizes reached to ~160 μm in diameter, as displayed in Figure 7g. Increasing the RBM time to 75 h led to an increase of the fresh surfaces of the unreacted Mg powders that consequently reacted with the hydrogen atoms to form MgH_2_ powders. Meanwhile, increasing the RBM time led to further powder refining, as indicated by the dramatic reduction in the powders particle size of the obtained after 75 h of RBM (less than 200 nm in diameter), as presented in Figure 7h or Figure 8a. These nanoscaled powder particles were aggregated to form microscaled particles (~2.3 μm in diameter) after 100 h of RBM, as presented in Figure 7i or Figure 8a. 

The XRD patterns of the powder particles obtained after 100 h of RBM revealed the formation of a single β-MgH_2_ phase coexisted with small molecular fractions of metastable of γ-MgH_2_ phase, as displayed in Figure 9b. It is worth noting that the Bragg-peaks related to unreacted Mg-metal had already disappeared, suggesting the completion of the RBM process (Figure 9b). The formation of nanoscaled MgH_2_ powder particles was confirmed by HRTEM, which indicated the formation of nano-dimensional particles with ~12 nm in diameter, as presented in Figure 9c. The filtered image taken for the zone shown in Figure 9c, displayed the lattice fringe image of tetragonal—MgH_2_ (β-phase)—in projection <110> direction (Figure 9d). The measured interplanar lattice -spacing distance (d), which was measured and found to be 0.3188 nm (Figure 9d) matched well with the reported value (0.3185 nm) of β-MgH_2_ (PDF file# 01-080-4431). Scanning transmission electron microscope, STEM (Figure 9e) was used together with X-ray EDS mapping technique to understand the distribution of elemental Ti-metal nanoparticles in the MgH_2_ matrix. 

The powder particles obtained at this final stage of RBM (100 h) possessed a fine structure beyond the sub-nano level (Figure 9e). The overall MgH_2_ matrix coexisted with ultrafine lenses of metallic-Ti particles (Figure 9f), which were homogeneously distributed in the matrix, as displayed in Figure 9g. The EDS analysis performed on 10 different samples show that the average composition of Ti- was 2.23 wt%.

#### 3.1.3. Color Changes with Changing the RBM Time 

**Figure 10:**
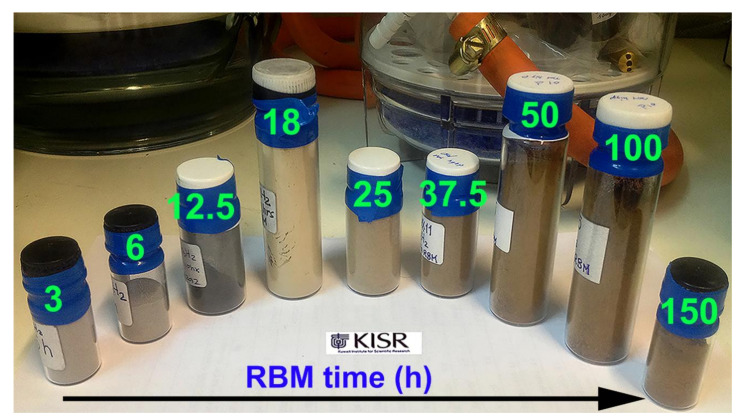
As—cold rolled Mg—shots milled for different RBM time (3, 6, 12.5, 18, 25, 37.5, 50, 100, and 150 h). The significant effect of RBM time on the samples’ color is notified.

The changes in the samples by visual inspection and discoloration were used conducted as a means to confirm the formation of the MgH_2_ phase. Figure 10 shows an optical image taken for the samples obtained after RBM at different times. 

At the early stage (3–6 h) of RBM, where the samples consisted of large powder particles (Figure 7d or Figure 8a), colors remained consistent when compared with the color of the feedstock (Figure 1i). The grayish color changed to darker shades upon RBM for 12.5 h (Figure 10), indicating the formation of finer powder particles (Figure 7e, and Figure 8a). Increasing the RBM time (18 h) enhanced the mechanically-induced gas-solid reaction, where a considerable volume fractions of MgH_2_ phase were obtained, as confirmed by the thermal analysis (Section 3.2.1.) and hydrogenation kinetics measurements (Section 3.3). Accordingly, the color of the sample changed from a dark –grey to light brown (beige), as shown in Figure 10. After 25 h of RBM, where the volume fraction of MgH_2_ was increased against the unreacted Mg-shots (Figure 9a) the color of the sample had a brown color, as shown in Figure 10.

The brown color of the samples was gradually changed into dark brown upon increasing the RBM time 37.5 and 50 h (Figure 10), implying an increase in volume fraction MgH_2_ powders and formation of finer particles. The powders obtained after 100–150 h of RBM, which revealed a tetragonal structure corresponding to the MgH_2_ phase (Figure 9b) had a dark brown color, as displayed in Figure 10. The changes from light –brown to dark -brown color can be used as a rough indication of formation fine nanopowder nanoparticles (Figure 1e, Figure 7i, or Figure 8a). 

### 3.2. Thermal Analysis

#### 3.2.1. Differential Scanning Calorimetry Measurements

**Figure 11:**
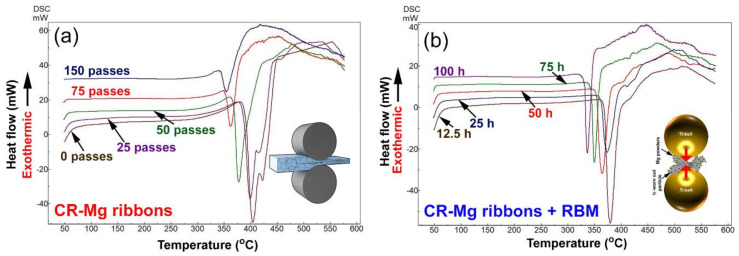
Differential scanning calorimeter (DSC) thermograms of Mg-ribbons (**a**) obtained after CR for different passes (0, 25, 50, 75, and 150 passes), and then hydrogenated at 300 °C, and (**b**) obtained after CR for 150 passes, and then RBM under hydrogen gas pressure for 12.5, 25, 50, 75, and 100 h of RBM time.

The thermal stability of all samples, characterized by the decomposition temperature (T^dec^) of MgH_2_ powders were investigated with DSC using a heating rate of 10 °C/min and a He gas flow of 50 mL/min. Figure 11a shows the DSC thermograms of Mg-strips that CR for 0, 25, 50, 75, and 150 passes before being hydrogenated at 300 °C. Meanwhile, the DSC traces for the as-CR Mg strips for 150 passes and then subjected to RBM under H_2_ gas pressure for 3, 12.5, 25, 50, and 100 h and are shown collectively in Figure 11b. 

All the MgH_2_ strips without exception revealed single endothermic events related to the decomposition of the MgH_2_ phase. The position of the endothermic peaks tended to shift upon increasing the CR passes, as suggested by the decrease in T^dec^ value of the MgH_2_ phase (Figure 11a). The starting MS -Mg ribbons (0 passes) displayed a high value of T^dec^ (404 °C), increasing CR passes to 50 and 75, which led to an outstanding decrease of T^dec^ to 377 °C and 362 °C, respectively (Figure 11a). Towards the end of CR processing (150 passes), the H_2_ was released at a relatively low T^dec^ of 354 °C. 

Elsewhere, the decomposition behavior of as-CR Mg-ribbons for 150 passes and then RBM for different RBM time, showed superior improvement with increasing the RBM time (Figure 11b). The T^dec^ of the sample milled for 12.5 h (379 °C) decreased to 374 °C and 364 °C after 25 h and 50, respectively (Figure 11b). Further improvement in T^dec^ was attained with increasing the RBM time to 75 h (351 °C) and 100 h (337 °C), as shown in Figure 11b. 

It can be concluded that the mechanical treatment of Mg-ribbons, using two consequent steps of CR and RBM techniques led to a superior improving of the decomposition characteristics of MgH_2_. We should emphases that when MgH_2_ powder was prepared upon RBM of Mg under pressurized H_2_ gas, the recorded value of T^dec^ was 399 °C [48]. Comparing that value with the one investigated in the present study (337 °C), we can claim that the CR process is an important primary step that should be employed prior to the RBM process. In addition to the importance of CR treatment, the degradation presented in the T^dec^ for MgH_2_ powders, could be partially attributed to the presence of significant molecular fractions (2.23 wt%) of Ti fine particles that were worn out from Ti-balls milling media and gradually introduced into the MgH_2_ matrix during the RBM process. It is worth noting that when the conventional tool-steel balls were used as milling media, the T^dec^ value of as-RBM MgH_2_ powders was 402 °C [49].

#### 3.2.2. Correlations between Processing Approaches, Grain Size and Decomposition Temperatures

**Figure 12:**
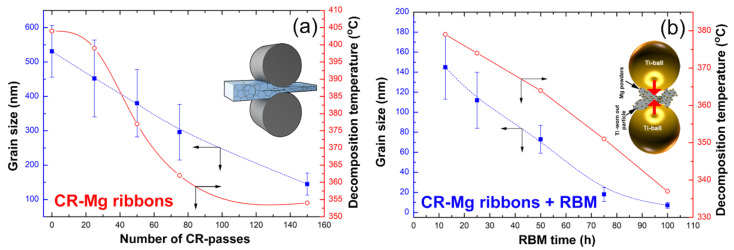
Correlations between processing approaches, grain sizes, and decomposition temperature for MgH_2_ prepared through (**a**) CR, and (**b**) CR +RBM techniques.

The influence of processing approaches (CR and CR +RBM), on the grain size, and T^dec^ for MgH_2_ has been investigated as a function of the processing time (CR passes and RBM time). The grain sizes of all samples were measured by the FE-HRTEM technique. The grain size of MgH_2_ prepared from the as-cast Mg ingot (0 passes) was 531 nm, where its corresponding T^dec^ was 404 °C, as shown in Figure 12a. When the sample was CR for 25 and 50 passes, their grains decreased significantly to 452 nm and 380 nm, respectively (Figure 12a). Accordingly, the corresponded T^dec^ for 25 passes and 50 passes samples were significantly decreased to reach 399 °C and 377 °C, respectively. The introduction of high-density imperfections (Figure 5a) through further CR passes (75 to 150 passes) disintegrated the Mg particles into finer nanosized grains, ranging between 296 nm to 145 nm, as shown in Figure 12a. The presence of these fine MgH_2_ minimized the H_2_ diffusion distance, leading to recorded T^dec^ for 75 passes and 150 passes samples were 362 °C and 354 °C, respectively (Figure 12a). 

The mechanically deformed Mg-ribbons obtained after 150 passes were subjected to different types of imperfections upon through RBM process. In this process, the materials were subjected to point and lattice defects, as well as severe dislocations [33,34,35,36,37,38,39,40,41,42,43,44,45,46,47,48,49], led to a dramatic reduction of the grain size. The results showed that both the grain size and T^dec^ were linearly decreased parallel to each other upon increasing the RBM time (Figure 12b). After 12.5 h of RBM time, the grain size of MgH_2_ powders was significantly reduced (145 nm), where the corresponding T^dec^ was measured to be 379 °C, as presented in Figure 12b. 

Increasing the RBM time (25 h to 50 h) led to a drastic decrease in the gain size of MgH_2_ (112 nm to 73 nm). This rapid decrease in the grain size improved the dehydrogenation process, as indicated by the rapid decrease in their T^dec^ reaching 374 °C and 364 °C, respectively (Figure 12b). Towards the end of RBM process (75 h to 100 h) the samples grain sizes were severely reduced to 18 nm and 7 nm in diameter (Figure 1e or Figure 9c), where their T^dec^ values reached to 351 °C and 337 °C, respectively (Figure 12b).

### 3.3. Hydrogenation/Dehydrogenation Kinetics

The effect of processing on the hydrogenation and dehydrogenation kinetics behavior of all samples was investigated as a function of processing time (CR-passes and RBM time). In these experiments, the samples were first activated at 350 °C under high H_2_ pressure of 35 bar for 4 h. The samples were then subjected to 100 continuous sorption/desorption cycles, conducted at 300 °C under H_2_ gas pressure fluctuated from 35 bar (absorption) to 400 mbar (desorption). The activation process is a necessary step to reduce the thin MgO layer coated the powder, which prevents the hydrogen gas to react with the fresh surfaces of Mg metals. In all experiments, the hydrogenation reactions were conducted at 275 °C under 10 bar of H_2_ gas pressure. However, the dehydrogenation kinetics were investigated at 275 °C under 400 mbar of hydrogen gas pressure. 

#### 3.3.1. CR-Mg Ribbons

**Figure 13:**
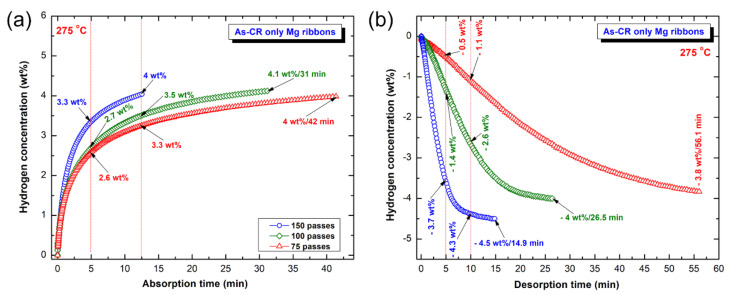
The kinetics of (**a**) hydrogenation, and (**b**) dehydrogenation measured at 275 °C for CR Mg-ribbons obtained after 75, 100 and 150 passes. The hydrogenation and consequent dehydrogenation processes were conducted under 10 bar and 400 mbar of H_2_ pressure, respectively.

Figure 13 displays the effect of RBM time on the hydrogenation (a) and dehydrogenation kinetics of as-CR Mg ribbons for different passes, ranging from 75 to 150 passes. All the CR samples revealed a good ability to absorb hydrogen at different time scales, as shown in Figure 13a. The samples that were subjected to CR of 75 and 100 passes, revealed slow kinetics, characterized by a low hydrogen concentration (2.6–2.7 wt%) absorbed after 5 min (Figure 13a). In contrast, the CR sample for 150 passes, was capable to absorb 3.3 wt% H_2_ after 5 min, as presented in Figure 13a. Increasing the applied absorption time to 12.5 min led to a remarkable increase in the hydrogen absorbed by the samples CR for 75 passes (3.3 wt%) and 100 passes (305 wt%), as displayed in Figure 13a. After this absorption time, the sample CR for 150 passes showed better hydrogenation kinetics, indicated by its highest hydrogen concentration value of 4 wt%, as shown in Figure 13a. Both of the samples, which were CR for 75 passes and 100 passes, required saturation for 42 min and 31 min to get saturation values of almost 4 wt% H_2_ (Figure 13a).

Likewise, the hydrogenation kinetics that was improved with increasing the number of CR passes applied to Mg-ribbons, the dehydrogenation kinetics of MgH_2_ behaved in the same manner. The CR samples for 75 and 100 passes revealed slow kinetics of hydrogen desorption, as suggested by their very low hydrogen concentration of −0.5 wt% and −1.4 wt% obtained after 5 min, respectively (Figure 13b). Their dehydrogenation kinetics were marginally improved upon increasing the applied desorption time (10 min), as suggested by increasing hydrogen concentration to −1.1 wt% and −2.6 wt%, respectively (Figure 13b). After very long desorption time (56.1 min) the hydrogen concentration of the sample CR for 75 passes reached −3.8 wt%, as displayed in Figure 13b. The CR sample for 100 passes showed better desorption kinetics, implied by the rather shorter time (26.5 min) required to desorb −4 wt% H_2_, as presented in Figure 13b. Superior Improvement in the dehydrogenation kinetics was attained upon CR the sample for 150 passes. This was implied by the sample capability to release −3.7 and 4.3 wt% H_2_ within 5 and 10 min, respectively (Figure 13b). The sample tended to desorb −4.5 wt% H_2_ after −14.9 min (Figure 13b). 

It can be then concluded that increasing the number of CR passes lead to a significant increase in the hydrogen storage capacity of Mg ribbons and enhanced their hydrogenation/dehydrogenation kinetics. The beneficial effect of CR on the hydrogen storage capacity and the kinetics behavior upon increasing the number of passes can be attributed to the dramatic reduction of crystallite size upon increasing the number of passes, as shown in Figure 12a. Furthermore, the presence of defects in Mg-ribbons upon severe plastic deformation conducted by CR that act as nucleation points for MgH_2_ is believed to improve the kinetics behavior of the metal hydride phase [50]. Unfortunately, increasing the CR passes of Mg-ribbons may lead to the formation of MgO layer coating the Mg-ribbon, as pointed out by Hout and Tousignant [50].

**Figure 14:**
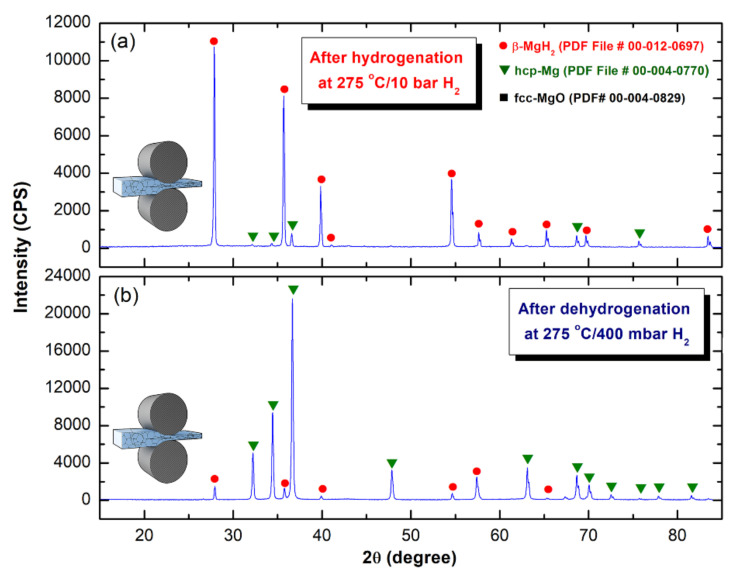
The XRD patterns of the 150 passes-sample obtained after (**a**) hydrogenation at 275 °C/10 bar H_2_, and (**b**) dehydrogenation at 275 °C/400 mbar H_2_.

Figure 14a shows the XRD pattern of as-CR sample for 150 passes and then hydrogenated at 275 °C/10 bar H_2_. The sample revealed a tetragonal-structure related to the formation of the β-MgH_2_ phase, as denoted by the red symbols shown in Figure 14a. The very small molar fraction of unreacted hcp- Mg shots exist in the sample (Figure 14a). This may be due to the short- hydrogenation time (12.5 min), applied for this sample (Figure 13a). The XRD pattern of the same sample obtained after dehydrogenation at 275 °C/400 mbar H_2_, is presented in Figure 14b. Those Bragg-peaks related to β-MgH_2_ phase (Figure 13a) were replaced by a set of polycrystalline hcp-Mg, as shown in Figure 14b. This implies the completion of the dehydrogenation process, where H_2_ gas was released from the sample. It should be noted that a very small mole fraction of undecomposed β-MgH_2_ is existed in the sample, as characterized by the low-intensity Bragg-peaks of β-MgH_2_ (Figure 14b). The presence of this undecomposed hydride phase may be attributed to the shortage of the desorption time applied for this sample (14.9 min). 

#### 3.3.2. RBM Mg Powders 

Despite the improvement in the H_2_ gas uptake/release kinetics achieved upon CR of Mg-ribbon, the obtained H_2_ storage capacity is modest, when compared with the theoretical hydrogen capacity of MgH_2_ [45]. In addition, the hydrogenation/dehydrogenation kinetics needs further improvement. These drawbacks of CR are mainly attributed to the disability of the process to produce nano-dimensional crystallites (under 100 nm) of MgH_2_ powders as presented in Figure 12a. Preparing MgH_2_ powders starting from pure Mg metal powders through the RBM technique is considered to be powerful tool to obtain nanocrystalline grains with sizes ranging between 8 nm to 15 nm in diameter [48,49]. 

In contrast to the beneficial size-reduction attained by RBM, the hydrogenation/dehydrogenation kinetics of the corresponding powders are rather poor [21,23,30,39] and required to be severely improved. One approach used to improve the kinetics behavior of RBM powders catalyzed the MgH_2_ with one or more catalytic agents, as discussed in the introduction. However, doping the hydride phase with catalysts leads to enhancing its kinetics behavior, it leads to a significant degradation in the hydrogen storage capacity of MgH_2_ [20]. Moreover, the RBM technique does not introduce a high density of plastic deformation to the milled powders, when compared with the CR process [41,50]. Here, we have tried to combine the beneficial effects of the three methods (e.g., CR, RBM, and catalyzation) together in one approach. For this purpose, the CR Mg-ribbons for 150 passes were used as feedstock materials for RBM, conducted under 15 bar of H_2_ gas pressure. Instead of using the common tool-steel balls, Ti-balls were employed as milling media. The purpose of using Ti-balls is to conduct in-situ catalyzation of MgH_2_ with Ti fine particles that worn from the balls during the milling process. 

**Figure 15:**
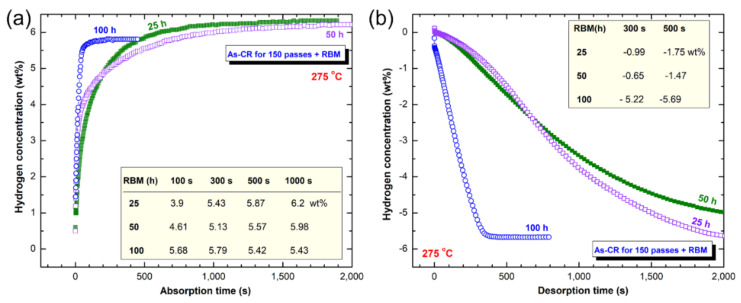
The kinetics of (**a**) hydrogenation, and (**b**) dehydrogenation measured at 275 °C for CR Mg-ribbons obtained after 150 passes, and then RBM for 25, 50, and 100 h. The hydrogenation and consequent dehydrogenation processes were conducted under 10 bar and 400 mbar of H_2_ pressure, respectively.

**Figure 16:**
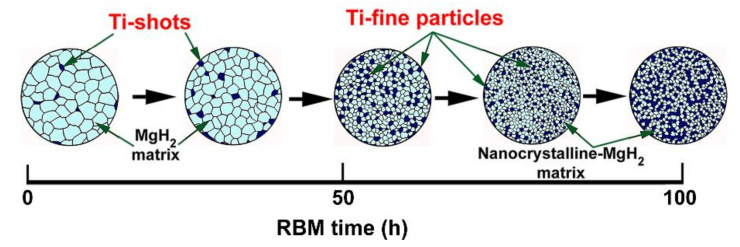
A Schematic presents the effect of RBM time in the reduction of MgH_2_ grain sizes. During the RBM process, Ti particles were worn out from the Ti-balls and gradually embedded into the MgH_2_ matrix. These spheroid particles that acted as nanocatalytic agent led to improve the hydrogenation/dehydrogenation kinetics of MgH_2_ nanopowders.

The hydrogenation/dehydrogenation kinetics behavior of CR Mg-ribbons investigated after 25, 50, and 100 h of RBM is shown in Figure 15a,b, respectively. At the early stage of RBM (25 h), the powders revealed slow hydrogenation kinetics at 275 °C, as characterized by the rather long time of 300 s (5 min) required to absorb 5.43 wt% H_2_), as presented in Figure 15a. Increasing the absorption time to 500 s (~8.3 min) and 1000 s (~16.7 min) led to a remarkable increase in the hydrogen storage capacity that reached to 5.87 and 6.2 wt.% H_2_, respectively (Figure 15a). The slow gas uptake kinetics was attributed to the morphological characteristics of the sample obtained after 25 h of RBM, which composited of large crystallites, and a small molecular fraction of Ti, as schematically presented in Figure 16. 

The hydrogenation kinetics behavior of the 50 h-RBM sample was very close to the sample obtained after 25 h of RBM. This is indicated by the close values of hydrogen storage and the absorption time required to get saturation, as shown in table inset of Figure 15a. It should be noted that the H_2_ storage capacity of the sample obtained after 50 h (5.98 wt%) is less than the one showed for 25 h (Figure 15a). This was attributed to the higher volume fraction of Ti (~1.42 wt%) introduced to the sample.

Increasing the RBM time (100 h) led to a decrease in the size for both of the MgH_2_ crystallites and Ti particles (Figure 16) upon introducing a high-density imperfection network in milled powder. As the crystallite size of the metal hydride decreased, the hydrogen diffusion distance was also decreased to facilitate faster kinetics [30]. This coupled with an increase in the molecular fraction of Ti–particles (2.23 wt%) worn out from the balls and embedded into the MgH_2_ matrix, as shown in Figure 16. The 100 h -RBM sample possessed superior absorption kinetics, indexed by a very short time of 100 s (~1.7 min) needed to absorb −5.68 wt% H_2_, as shown in Figure 15a. After 300 s (5 min), the sample was almost saturated at 5.79 wt% H_2_ (Figure 15a). The dehydrogenation kinetics of as-CR Mg-ribbons for 150 passes that were RBM for different time (25, 50, and 100 h) are presented in Figure 15b. Those samples obtained after 25 and 50 h of RBM possessed poor desorption kinetics, characterized by a long time of 2000 s (~33.3 min) necessary to release −1.75 and −1.47 wt% H_2_, respectively (Figure 15b). In contrast, the sample, which was obtained after longer RBM time (100 h) revealed superior desorption behavior, characterized by a short time of 500 s (~8.3 min) consumed to release 5.69 wt% H_2_, as displayed in Figure 15b.

**Figure 17:**
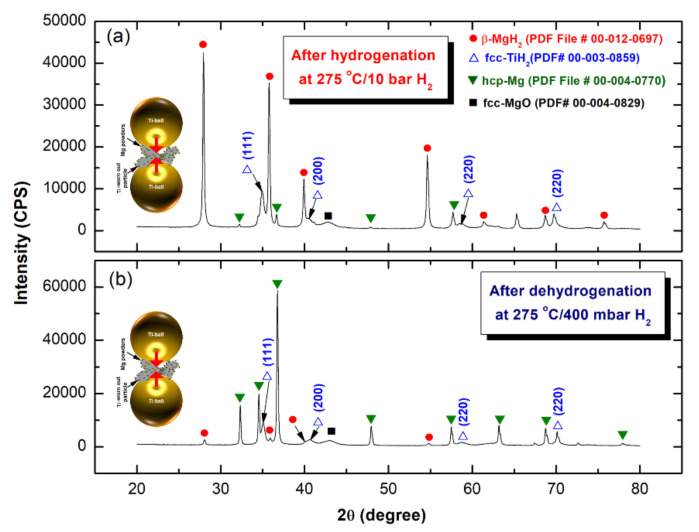
The XRD patterns of the 150 passes CR Mg-ribbons that were RBM for 100 h and then (**a**) hydrogenated at 275 °C/10 bar H_2_, and (**b**) dehydrogenated at 275 °C/400 mbar H_2_.

Figure 17a shows the XRD pattern of the hydrogenated (275 °C/10 bar H_2_) 150-passes CR sample that was RBM for 100 h. The sample possessed a crystal structure of the tetragonal-structure of β-MgH_2_, as denoted by the red symbols shown in Figure 17a. However, the hydrogenation time applied for this sample was 400 s (Figure 15a), a very small volume fraction of unreacted Mg powders still existed in the sample obtained after the hydrogenation process. This was suggested by those Bragg lines related to hcp-Mg crystals displayed in Figure 17a. Probably, some of pure Mg powders were adhered to the internal wall of the Cu-sample holder that came out with the hydrogenated powders upon discharging the powder. Meanwhile, the XRD pattern of the same sample obtained after dehydrogenation at 275 °C/400 mbar H_2_, is presented in Figure 17b. The Bragg-peaks related to β-MgH_2_ phase (Figure 17a) were replaced by a set of polycrystalline hcp-Mg, as shown in Figure 17b. This implying the completion of the dehydrogenation process, and formation of Mg powders that were coexisted with a very small volume fraction of undecomposed β-MgH_2_ phase, as shown in Figure 17b.

#### 3.3.3. Cycle Lifetime Test

The capability of nanocomposite the powders obtained after 150 h of RBM to achieve continuous cyclic hydrogenation/dehydrogenation processes was investigated at 225 °C. This test is necessary to realize the ability of the fabricated nanocomposite system on maintaining their hydrogen storage capacity and fast hydrogenation/dehydrogenation kinetic without serious degradation or failure. In the present study, a small amount (~350 mg) of the powders was continuously subjected to hydrogenation/dehydrogenation cycles conducted at 275 °C under a hydrogen gas pressure of 10 bar (hydrogenation) 400 mbar (dehydrogenation) for 200 h. In this experiment, the powders were firstly activated with applying cyclic hydrogen gas sorption/desorption under a pressure of 35 bar at 350 °C for 30 h. This treatment was necessary for the surface cleaning of the powders and to break down the oxide film (MgO) developed on the powder surfaces during handling the sample outside of the glove box. 

**Figure 18:**
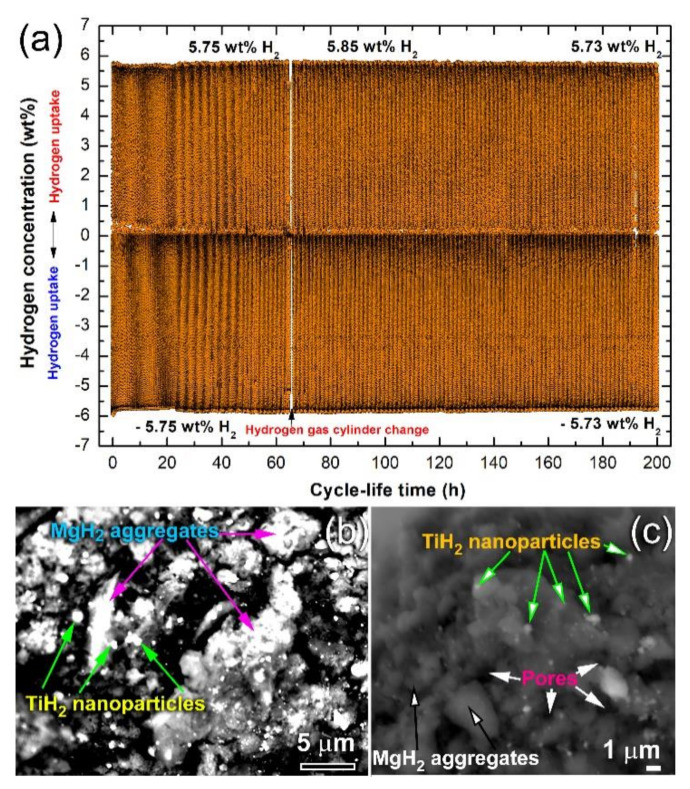
(**a**) Cycle-life-time curves of the 150 passes -CR sample, which was RBM for 150 h. The FE-backscattered electrons (BSE) micrographs of the samples taken after 199.5 h hydrogenation and 200 h dehydrogenation time are presented in (**b**), and (**c**), respectively. The H_2_-uptake and release were conducted at 275 ^°^C/10 bar H_2_, and 275 °C/400 mbar H_2_, respectively.

Figure 18a shows the hydrogen absorbed/desorbed cycles achieved continuously for 200 h at a temperature of 275 °C. This system possessed an excellent hydrogenation/dehydrogenation cyclability, demonstrated by achieving continuous 320 cycles within 200 h without failure (Figure 18a). No serious degradation in the hydrogen storage capacity, which was in the range between 5.73–5.85 wt%, could be detected even after 200 h, as shown in Figure 18a. Moreover, the kinetics of the hydrogenation/dehydrogenation processes remaining nearly constant without failure or serious decay. It can be noticed that doping MgH_2_ with Ti nanoparticles that worn for the Ti-balls during RBM allowed the catalytic agent particles to occupy the surface of MgH_2_ powders with uniform distribution, as shown in Figure 18a. During the hydrogenation process, all the Ti nanoparticles reacted with H_2_ gas at 275 °C/10 bar H_2_ to form TiH_2_ nanopowders that were uniformly distributed in the MgH_2_ matrix, as shown in Figure 18b. 

## 4. Conclusions

Utilization of solid waste (SW) Mg metal for tailoring high-hydrogen storage capacity nanoparticles has never been reported as yet. The present study demonstrated an effective multistage process used to fabricate high-grade of Mg metal, stating from Mg machining chips. Melting/casting, melt spinning (MS), cold rolling (CR) were dedicated to producing purity Mg strips (99.88 wt%). The recycled Mg-metal was used as feedstock materials for synthesizing nanocrystalline MgH_2_ powders through a reactive ball milling (RBM) approach, using a tumbler ball mill pressurized with 15 bar of H_2_ gas. The following main conclusions could be drawn from this investigation:

- MS technique is an ideal technique used to produce thin Mg-ribbons.
- CR process is a vital step used to introduce severe plastic deformation in Mg-ribbons, resulting in the formation of fine crystallites.
- Increasing the number of CR passes led to a significant reduction in the Mg crystallites. Accordingly, this led to an increase in the hydrogen storage capacity of metallic Mg and enhance the hydrogenation/dehydrogenation kinetics.
- Further increase in the hydrogen storage capacity and superior improvement in the gas uptake/release kinetics can be attained upon ball milling the as-CR Mg ribbons under high H_2_ pressure.
- Using Ti-balls milling media had obvious benefits for improving the hydrogenation/dehydrogenation processes that were taken place very fast when compared with MgH_2_ powders milled with tool steel-balls.
